# Efficacy of intraovarian autologous platelet-rich plasma in women with poor ovarian reserve or ovarian insufficiency: a meta-analysis of randomized controlled trials

**DOI:** 10.3389/fendo.2026.1827041

**Published:** 2026-05-12

**Authors:** Yuan Fang, Chunhua Liu, Zixin He, Yongfei Zheng, Ling Zhu, Zhi Ye

**Affiliations:** 1 Lishui Hospital of Traditional Chinese Medicine Affiliated to Zhejiang University of Chinese Medicine, Lishui, Zhejiang, China; 2 Department of Gynecology, First Affiliated Hospital, Guangzhou University of Chinese Medicine, Guangzhou, Guangdong, China

**Keywords:** autologous platelet-rich plasma, meta-analysis, platelet-rich plasma, poor ovarian reserve, PRP

## Abstract

**Background:**

Poor ovarian reserve and ovarian insufficiency pose major challenges in reproductive medicine, with limited effective therapeutic options. Intraovarian autologous platelet-rich plasma (PRP) has shown potential benefits, but its efficacy remains uncertain based on current randomized controlled trials (RCTs).

**Methods:**

This review followed PRISMA guidelines and was registered in PROSPERO (CRD420251233432). We systematically searched PubMed, Cochrane Central, Embase, CNKI, VIP, and Wanfang up to November 22, 2025, for randomized controlled trials of intraovarian autologous PRP in women with diminished ovarian reserve or ovarian insufficiency. Meta-analyses were performed using Stata 16, with heterogeneity assessed by the I² statistic. Risk of bias and study quality were evaluated using the Cochrane RoB 2 tool and the PEDro scale. Primary outcomes included serum anti-Müllerian hormone (AMH), follicle-stimulating hormone (FSH), antral follicle count (AFC), and pregnancy rates.

**Result(s):**

This meta-analysis included seven RCTs involving 422 women with poor ovarian reserve or ovarian insufficiency/failure. PRP treatment was associated with significant improvements in several ovarian reserve-related parameters: AFC increased (MD = 0.81, 95% CI 0.31 to 1.30; I² = 41.63%), AMH increased (SMD = 0.91, 95% CI 0.41 to 1.42; I² = 54.32%), FSH decreased (MD = -5.72, 95% CI -7.03 to -4.41; I² = 0.00%), and estradiol increased (MD = 26.03, 95% CI 19.53 to 32.53; I² = 0.22%). However, PRP treatment did not significantly improve pregnancy rates compared with controls.

**Conclusions:**

This meta-analysis suggests that intraovarian PRP may improve ovarian reserve-related markers in women with poor ovarian reserve or ovarian insufficiency/failure, but it does not appear to increase pregnancy rates significantly. These findings should be interpreted cautiously because of the limited number of studies and heterogeneity in treatment protocols, and current evidence remains insufficient to define the optimal PRP regimen or the patient subgroups most likely to benefit.

**Systematic Review Registration:**

https://www.crd.york.ac.uk/PROSPERO/view/CRD420251233432, identifier CRD420251233432.

## 1 Introduction

Diminished ovarian reserve is a major contributor to female infertility, characterized by a reduced pool of recruitable follicles and impaired ovarian endocrine function (1). It commonly presents as diminished ovarian reserve (DOR) or premature ovarian insufficiency (POI) (2, 3). With the trend toward delayed childbearing, the proportion of such patients in reproductive centers continues to rise. These conditions typically feature lower antral follicle counts, reduced AMH levels, elevated FSH concentrations, and overall reduced reproductive potential, resulting in a poor ovarian response, fewer retrieved oocytes, and lower pregnancy rates (4, 5).

Women with DOR experience a progressive decline in both follicular quantity and oocyte competence, which directly limits their response to ovarian stimulation and overall reproductive potential (6). In more severe forms, such as premature ovarian insufficiency, ovarian endocrine activity becomes increasingly unstable, leading to unpredictable follicular development and markedly reduced fertility prospects (7, 8). Current therapeutic approaches focus on optimizing stimulation strategies and using adjunctive agents, such as DHEA, coenzyme Q10, growth hormone, and antioxidant supplements; however, reported benefits are modest and often inconsistent (9, 10). In POI, hormone replacement therapy primarily mitigates hypoestrogenic symptoms but offers minimal improvement in ovarian reserve or fertility outcomes (11). Emerging regenerative options, including ovarian tissue activation and stem cell–based interventions, have demonstrated encouraging preliminary findings, but these techniques remain experimental with no robust randomized trials to support their efficacy (12, 13). Overall, available treatments provide limited and variable benefits and do not address the underlying deterioration of ovarian function, underscoring the need for innovative approaches that can restore the ovarian microenvironment and enhance follicular activity.

Platelet-rich plasma (PRP) is an autologous biologic concentrate obtained through centrifugation of peripheral blood, characterized by platelet levels several times above baseline and the release of multiple growth factors from α-granules, including PDGF, TGF-β, VEGF, and IGF-1 (14, 15). These bioactive molecules play essential roles in tissue repair, angiogenesis, and modulation of the local microenvironment (16). Owing to its regenerative and anti-inflammatory properties, PRP has been widely applied in orthopedics, dermatology, sports medicine, ophthalmology, and aesthetic medicine, with substantial evidence supporting its safety (17, 18). In recent years, PRP has been introduced into gynecology and reproductive medicine, where emerging experimental studies suggest it may enhance ovarian blood flow, stimulate growth-factor signaling, and modulate endocrine and paracrine pathways, ultimately creating a more favorable milieu for follicular development (19, 20). Building on these potential mechanisms, the use of PRP in women with DOR, POI, or POR has gained increasing attention. Several studies have reported improvements following intraovarian PRP administration, including increases in antral follicle count, enhanced hormonal profiles, resumption of ovulation, and even spontaneous pregnancies (19, 21). However, most available evidence comes from small, single-center, non-randomized observational or prospective studies, which generally have low methodological quality (22, 23). Although recent systematic reviews and meta-analyses have attempted to summarize the therapeutic potential of PRP for ovarian dysfunction, their conclusions are based solely on non-RCT data, which are subject to substantial selection bias, limited control groups, and considerable heterogeneity (20, 21). As a result, the current findings remain inconsistent and insufficient to establish clear clinical recommendations regarding the efficacy and safety of PRP. Therefore, a rigorously conducted systematic review and meta-analysis of randomized controlled trials is needed to better evaluate the clinical value of PRP in women with impaired ovarian reserve and to inform future high-quality trials.

## 2 Methods

### 2.1 Registration and reporting standards

This systematic review and meta-analysis followed the PRISMA-NMA reporting guidelines. The protocol was prospectively registered in PROSPERO (CRD420251233432). As this study involved the synthesis of published data, ethical approval and informed consent were not necessary.

### 2.2 Search strategies

This systematic review and meta-analysis evaluated the effects of PRP on ovarian reserve, including only RCTs. We searched PubMed, Cochrane Library, Embase, CNKI, Wanfang, and VIP for studies published up to November 22, 2025. The search combined MeSH terms and keywords with Boolean operators, covering “platelet-rich plasma,” “PRP,” and ovarian reserve–related conditions such as “diminished ovarian reserve,” “premature ovarian insufficiency/failure (POI/POF),” and “poor ovarian response (POR).” Terms were expanded to capture all relevant studies, given that reduced ovarian reserve underlies POR. No language restrictions were applied. Only RCTs meeting the inclusion criteria were analyzed. The full strategy is shown in **Supplementary Table 1**.

### 2.3 Inclusion criteria and exclusion criteria

#### 2.3.1 Inclusion criteria (based on the PICOS framework)

Participants (P): Women with DOR, POI, or POR.
Intervention (I): Intraovarian injection of PRP.
Comparison (C): Patients receiving standard ovarian stimulation without PRP.
Outcomes (O): Primary outcomes included serum anti-Müllerian hormone (AMH), follicle-stimulating hormone (FSH), estradiol (E2), and antral follicle count (AFC); secondary outcomes included ovarian stimulation response, number of oocytes retrieved, number of mature oocytes (MII), number of high-quality embryos, and pregnancy rate.
Study Design (S): RCTs only.

#### 2.3.2 The exclusion criteria were as follows

① Studies were excluded if they were non-original publications such as case reports, letters, conference abstracts, reviews, or commentaries;
② Lacked sufficient data to calculate treatment effects or safety outcomes (e.g., mean differences, relative risks, or 95% confidence intervals);
③ Did not report outcomes related to ovarian reserve or pregnancy after PRP treatment;
④ Involved duplicate data or failed to clearly define diagnostic criteria for diminished ovarian reserve, POI, or POR.

### 2.4 Literature screening and data extraction

Two authors (C.L. and Y.F.) independently screened all retrieved records using the predefined eligibility criteria. Citations were managed and duplicates removed with EndNote (Clarivate Analytics) before screening. Titles and abstracts were initially assessed to identify potentially eligible studies, followed by full-text review for final inclusion. Discrepancies between reviewers were resolved through discussion, with a third reviewer (Z.Y.) consulted if consensus could not be reached.

A structured data extraction form was used to collect key information from each included study, including first author, publication year, study location, design characteristics, diagnostic criteria, sample size, details of interventions and comparators, primary outcomes, and follow-up duration. For studies using PRP, additional details such as injection dose, site, treatment course, and frequency were recorded. Extracted outcomes included AMH, FSH, AFC, E2, and pregnancy rate. If standard deviations (SDs) were not reported, they were calculated from reported standard errors (SEs) or 95% confidence intervals following the Cochrane Handbook; when unavailable, SDs were estimated from ranges or interquartile ranges using the method described by Wan et al. (24, 25). When necessary data could not be obtained from the publications, the original study authors were contacted.

Data extraction was performed independently by two reviewers (C.L. and Y.F.) and subsequently cross-checked for consistency. Discrepancies in coding or interpretation were resolved through discussion, with a third reviewer consulted if consensus could not be reached. Inter-rater agreement was assessed using Cohen’s κ statistic, yielding values of 0.86 for title and abstract screening, 0.83 for full-text evaluation, and 0.84 for data extraction, indicating a high level of concordance between reviewers.

### 2.5 Assessment of risk of bias

The quality of the included studies was assessed using the Cochrane Risk of Bias 2 (RoB 2) tool, which evaluates bias related to the randomization process, deviations from intended interventions, missing outcome data, outcome measurement, and selective reporting. An overall risk rating was assigned to each trial. Two reviewers (Z.H. and Y.Z.) conducted the assessments independently and cross-checked their evaluations, with a third reviewer (Z.Y.) resolving any disagreements. Additionally, study quality was further evaluated using the Physiotherapy Evidence Database (PEDro) scale.

### 2.6 Subgroup analyses

To explore potential sources of heterogeneity, subgroup analyses were conducted according to ovarian condition and treatment-related characteristics when sufficient data were available. Because POF is an older term that largely overlaps with POI, studies involving these populations were combined into a POI/POF subgroup for the primary subgroup analysis. After re-examining the included studies, subgroup analysis was feasible only according to ovarian condition. Owing to the limited number of eligible studies and insufficient or inconsistently reported data within individual subgroups, formal subgroup analyses for other clinically relevant variables, including patient age, PRP preparation parameters, injection dose, injection frequency, duration of intervention, timing of intervention, and adjunctive treatments, were not performed.

### 2.7 Certainty of evidence assessment

The certainty of evidence for each primary outcome was evaluated using the GRADE approach via GRADEpro GDT software. This method assesses five domains affecting confidence in pooled results: study limitations, inconsistency, directness, precision, and publication bias. Since all included studies were RCTs, outcomes were initially rated as high certainty, with downgrading applied only when notable concerns arose. The final evidence ratings and synthesized results were summarized in a GRADEpro-generated Summary of Findings table.

### 2.8 Data synthesis and statistical analysis

Statistical analyses were conducted using RevMan 5.4. For continuous outcomes, effect sizes were calculated from the post-intervention means, standard deviations, and sample sizes of the intervention and control groups. Depending on whether the same or different measurement scales were used, results were expressed as mean differences (MDs) or standardized mean differences (SMDs), with 95% confidence intervals (CIs). For dichotomous outcomes, effect estimates were expressed as log risk ratios (LogRRs) with 95% CIs. Forest plots were generated to visualize the pooled estimates, and a two-sided P value < 0.05 was considered statistically significant.

Heterogeneity among studies was evaluated using Cochran’s Q test and the I² statistic. A fixed-effect model was applied when heterogeneity was low (I² < 50%), whereas a random-effects model was used when heterogeneity was substantial (I² ≥ 50%). Publication bias was not assessed because the number of included studies for each outcome was too small to allow meaningful evaluation.

## 3 Results

### 3.1 Search results and research selection

**Figure 1:**
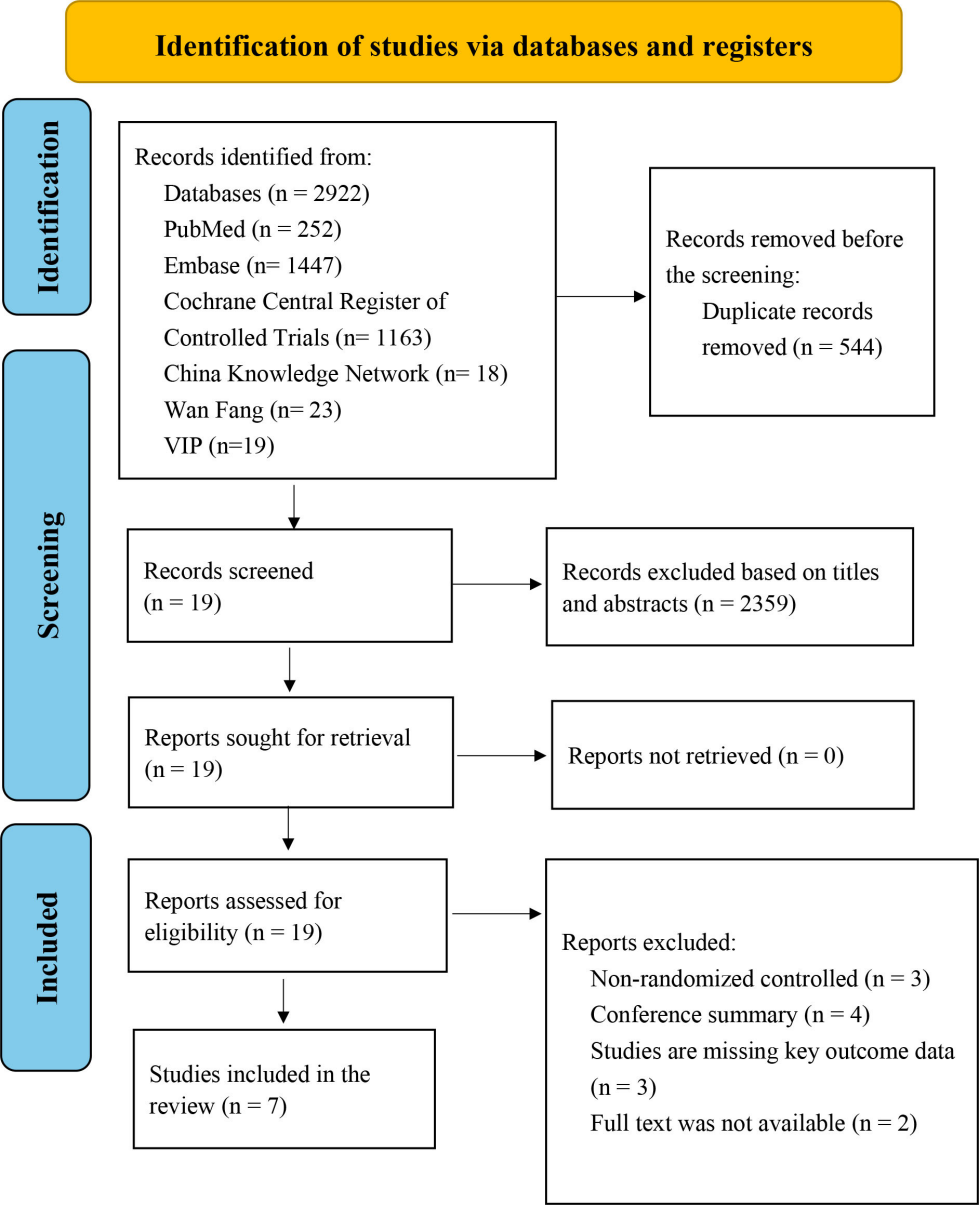
PRISMA flow diagram of study identification, screening, eligibility assessment, and inclusion.

As shown in **Figure 1**, the literature search initially identified 2,922 potentially relevant records. After removal of 544 duplicates, 2,378 records were screened based on titles and abstracts, and 19 articles were assessed for full-text eligibility. After detailed evaluation and reference screening, 12 studies were excluded for the following reasons: non-randomized design (n = 3), conference abstract only (n = 4), insufficient primary outcome data (n = 3), and unavailable full text (n = 2). Ultimately, seven RCTs were included in this review.

### 3.2 Study characteristics

**Table 1** summarizes the key characteristics of the included studies. A total of seven studies involving 422 women were analyzed, including one study on POR patients (29), three on POI patients (30–32), two on women with premature ovarian failure (POF) (26, 28), and one on DOR patients (27). The studies were conducted in Iran (n = 1), China (n = 3), the United States (n = 1), and Spain (n = 2). The volume of PRP injected ranged from 1 to 2 mL, and the timing of injection was randomized for all women with amenorrhea or ovulatory dysfunction. All injections were performed under ultrasound guidance via the transvaginal route, with laparoscopic injection used for ovaries that were not easily accessible.

**Table 1:** Baseline demographic and clinical characteristics of participants in the included studies.

Study	Country	Diagnostic	Design	PRP group						Control group			Outcome	Measurement timepoint (week)
				Sample size	Age (year)	Centrifugation spinning	Activator	Injections (N)/ volume (mL)	Injection location	Sample size	Age (year)	Intervention		
Chen Q, Zeng L, Lin W, et al. (26)	China	Premature Ovarian Failure	RCT, 2 arms	32	30.69 ± 5.04	Double	CaCl_2_	1/1	Both ovaries	32	31.56 ± 4.22	Hormone replacement therapy	Estradiol (E2), FSH, LH	12 weeks
Peng A, Ai M, Tan X, et al. (27)a	China	Diminished ovarian reserve	RCT, 4 arms	21	38.80 ± 4.50	Double	CaCl_2_	1/1.5	Both ovaries	7	36.20 ± 5.60	Blank control	FSH, AFC Pregnancy outcomes	12 weeks
Peng A, Ai M, Tan X, et al. (27)b	China	Diminished ovarian reserve	RCT, 4 arms	15	38.90 ± 3.60	Double	CaCl_2_	1/1.5	Both ovaries	8	36.20 ± 5.60	Blank control	FSH, AFC Pregnancy outcomes	12 weeks
Wang X (28).	China	Premature Ovarian Failure	RCT, 2 arms	45	35.16 ± 3.42	Double	Thrombin	1/1	Both ovaries	44	35.42 ± 3.17	Conventional medical treatment	FSH Estradiol (E2)	12 weeks
Barrenetxea G, Celis R, et al. (29)	Spain	Poor ovarian reserve	RCT, 2 arms	30	37.27 ± 0.52	Double	CaCl_2_	1/2	Both ovaries	29	37.91 ± 0.65	Conventional medical treatment	AFC, AMH Pregnancy outcomes	4 weeks
Herlihy N S, Cakiroglu Y, et al. (30)	USA	Primary ovarian insufficiency	RCT, 2 arms	41	34.4 ± 2.8	Double	CaCl_2_	1/1.5	Both ovaries	42	34.7 ± 2.1	Conventional medical treatment	FSH, AFC Estradiol (E2), AMH	4 weeks
Navarro C, Cabrera P T, Garrán A T (31).	Spain	Primary ovarian insufficiency	RCT, 3 arms	10	41.20 ± 2.86	Double	Thrombin	1/1	Both ovaries	10	41.20 ± 2.86	Physiological solution	FSH, AFC, AMH	4 weeks
Tehraninejad E S, Razavi M O, et al. (32)	Iran	Primary ovarian insufficiency	RCT, 2 arms	34	39.34 ± 3.88	Double	CaCl_2_	1/1.5	Both ovaries	22	40.81 ± 3.68	Blank control	FSH, AFC, AMH	4 weeks

Values are presented as mean ± standard deviation or the number of patients; RCT, Randomized controlled trial; PRP, platelet-rich plasma; AFC, Antral Follicle Count; AMH, Anti-Mullerian Hormone; Follicle FSH, Stimulating Hormone; LH, Luteinizing Hormone; E2, estradiol.

The overall characteristics and intervention details of the included studies are presented in **Table 1**. Additional baseline clinical characteristics of the participants, including duration of infertility and, where reported, baseline ovarian reserve-related markers, are summarized in **Table 2**.

**Table 2:** Baseline clinical characteristics of participants in the included studies.

Study	Duration of infertility (months)	PRP group				Control group			
		Baseline AMH	Baseline AFC	Baseline FSH	Baseline E2	Baseline AMH	Baseline AFC	Baseline FSH	Baseline E2
Chen Q, Zeng L, Lin W, et al. (26)	NR	NR	NR	15.12 ± 3.52	41.25 ± 14.85	NR	NR	10.58 ± 3.91	43.54 ± 15.25
Peng A, Ai M, Tan X, et al. (27)	18.18 ± 3.51	NR	2.38 ± 1.56	10.97 ± 4.45	NR	NR	2.00 ± 1.96	10.62 ± 4.41	NR
Peng A, Ai M, Tan X, et al. (27)	16.27 ± 3.43	NR	2.00 ± 0.74	13.35 ± 4.46	NR	NR	2.00 ± 1.96	10.62 ± 4.41	NR
Wang X (28).	16.18 ± 2.51	NR	NR	16.08 ± 5.09	57.84 ± 6.75	NR	NR	14.36 ± 4.12	59.11 ± 7.26
Barrenetxea G, Celis R, et al. (29)	NR	0.76 ± 0.45	3.88 ± 1.78	NR	NR	0.65 ± 0.58	3.92 ± 1.01	NR	NR
Herlihy N S, Cakiroglu Y, et al. (30)	NR	0.73 ± 0.46	5.20 ± 3.20	11.9 ± 6.30	NR	0.70 ± 0.46	5.60 ± 3.30	12.12 ± 5.32	NR
Navarro C, Cabrera P T, Garrán A T (31).	20.12 ± 5.24	0.56 ± 0.25	4.40 ± 1.50	25.4 ± 13.48	54.55 ± 15.46	0.50 ± 0.38	3.5 ± 1.27	23.8 ± 12.13	76.58 ± 16.56
Tehraninejad E S, Razavi M O, et al. (32)	NR	0.62 ± 0.49	4.14 ± 1.79	12.12 ± 6.80	NR	0.76 ± 0.40	4.09 ± 1.79	15.54 ± 6.36	NR

AMH, anti-Müllerian hormone (ng/mL); AFC, antral follicle count (count); FSH, follicle-stimulating hormone (IU/L); E2, estradiol (pg/mL); NR, not reported.

Data are presented as mean ± SD, where available.

### 3.3 Risk of bias

**Figure 2:**
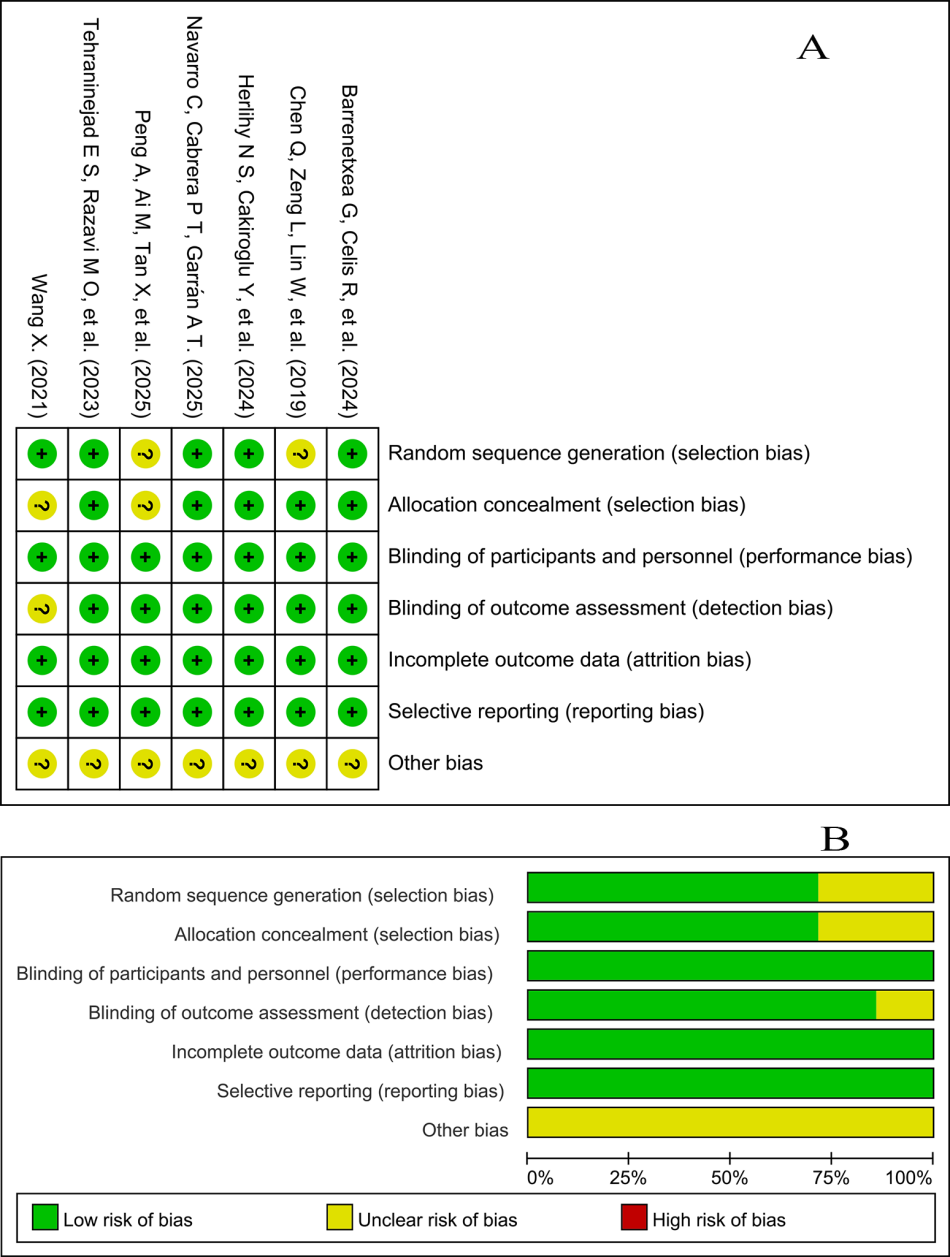
Risk-of-bias assessment of the included RCTs. **(A)** Risk-of-bias summary; **(B)** risk-of-bias graph. “+” indicates low risk of bias; “?” indicates unclear risk of bias; “−” indicates high risk of bias.

The methodological quality of the seven included RCTs was assessed independently by two reviewers using the Cochrane Risk of Bias 2 (RoB 2) tool, and the results are presented in **Figure 2**. Overall, most studies were judged to have a low risk of bias, although some showed minor concerns, mainly related to unclear allocation concealment.

### 3.4 Synthesis of results

#### 3.4.1 Effects of PRP on antral follicle count

**Figure 3:**
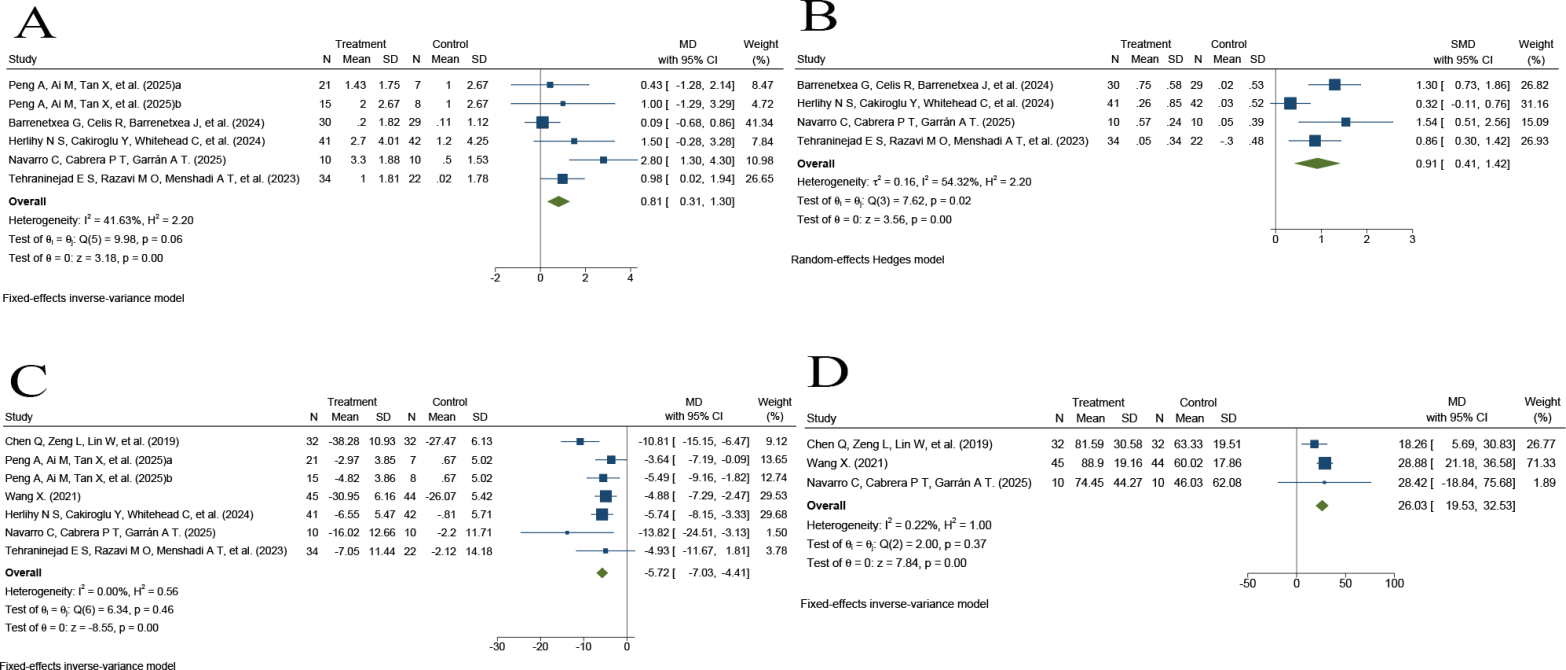
Forest plots of the pooled effects of intraovarian PRP injection on ovarian reserve-related markers in the included RCTs. **(A)** Antral follicle count (AFC); **(B)** anti-Müllerian hormone (AMH, ng/mL); **(C)** follicle-stimulating hormone (FSH, IU/L); **(D)** estradiol (E2, pg/mL). Effect estimates are presented as mean differences (MDs) or standardized mean differences (SMDs), as appropriate, with 95% confidence intervals (CIs).

Five RCTs (27, 29–32) involving 269 participants reported changes in AFC. The pooled analysis showed that AFC was significantly higher in the PRP group than in the control group (MD = 0.81, 95% CI 0.31 to 1.30, P < 0.001). No significant heterogeneity was observed among the studies (I² = 41.63%, P for heterogeneity = 0.06), indicating relatively consistent findings across trials (**Figure 3A**).

#### 3.4.2 Effects of PRP on anti-Müllerian hormone

Four RCTs (29–32) involving 218 participants reported changes in AMH levels. The pooled analysis showed that AMH was significantly higher in the PRP group than in the control group (SMD = 0.91, 95% CI 0.41 to 1.42, P < 0.001). Moderate heterogeneity was observed among the studies (I² = 54.32%, P for heterogeneity = 0.02), suggesting some between-study variability (**Figure 3B**).

#### 3.4.3 Effects of PRP on follicle stimulating hormone

Six RCTs (26–28, 30–32) involving 363 participants reported changes in FSH levels. The pooled analysis showed that FSH was significantly lower in the PRP group than in the control group (MD = -5.72, 95% CI -7.03 to -4.41, P < 0.001). No heterogeneity was detected among the included studies (I² = 0.00%, P for heterogeneity = 0.46), indicating highly consistent findings (**Figure 3C**).

#### 3.4.4 Effects of PRP on estradiol

Three RCTs (26, 28, 31) involving 173 participants reported changes in E2 levels. The pooled analysis showed that E2 was significantly higher in the PRP group than in the control group (MD = 26.03, 95% CI 19.53 to 32.53, P < 0.001). No significant heterogeneity was detected among the studies (I² = 0.22%, P for heterogeneity = 0.37), indicating highly consistent findings (**Figure 3D**).

### 3.5 Effects of PRP on pregnancy outcomes

**Figure 4:**
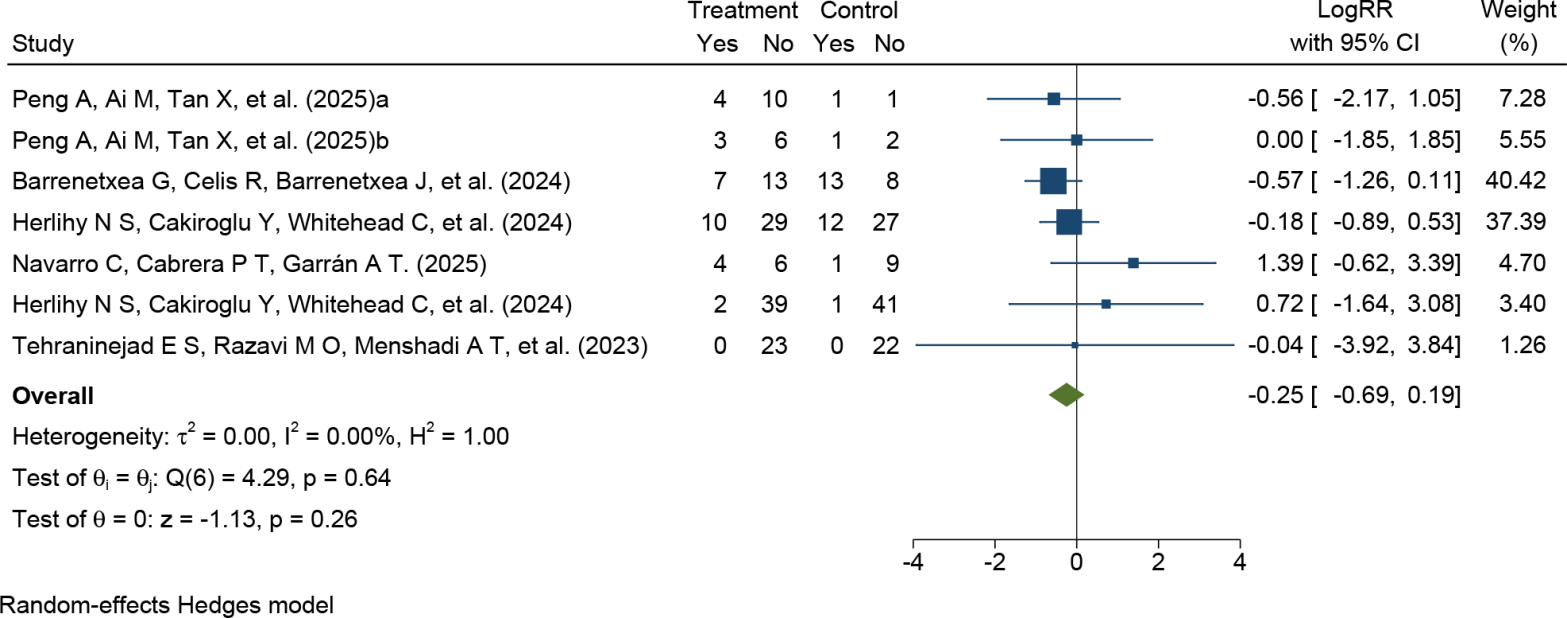
Forest plot of the pooled effect of intraovarian PRP treatment on clinical pregnancy rate in the included RCTs. Effect estimates are presented as log risk ratios (LogRRs) with 95% confidence intervals (CIs).

Six RCTs (27, 29–32) involving 228 participants reported pregnancy outcomes. The pooled analysis showed no significant difference in pregnancy rates between the PRP and control groups (LogRR = -0.25, 95% CI -0.69 to 0.19, P = 0.26). No heterogeneity was detected across the studies (I² = 0.00%, P for heterogeneity = 0.64), indicating consistent findings (**Figure 4**).

### 3.6 Subgroup analyses

Subgroup analyses were performed according to ovarian condition (POR vs POI/POF) to further explore potential clinical heterogeneity, and the corresponding results are shown in **Table 3**.

**Table 3:** Subgroup meta-analysis of intraovarian PRP treatment in women with poor ovarian reserve or ovarian insufficiency/failure.

Factor	No. of studies	Participants	Summar of MD/logRR	95% CI of MD/logRR	P	Heterogeneity (I^2^)	Model used
Poor Ovarian Reserve Subgroup							
Antral Follicle Count (count)	2	110	0.23	0.16 to 0.49	< 0.001	0.00%	Fixed
Anti-Mullerian Hormone (ng/mL)	1	59	0.63	0.45 to 0.87	< 0.001	NA	NA
Follicle Stimulating Hormone (IU/L)	1	51	-4.53	-6.09 to -1.98	< 0.001	NA	NA
Pregnancy Outcomes (count)	2	110	-0.45	-1.97 to 0.18	0.09	46.46%	Random
Primary Ovarian Insufficiency/Failure Subgroup							
Antral Follicle Count (count)	3	159	1.15	0.77 to 2.05	< 0.001	30.52%	Fixed
Anti-Mullerian Hormone (ng/mL)	3	159	0.37	0.22 to 0.52	< 0.001	0.00%	Fixed
Follicle Stimulating Hormone (IU/L)	4	248	-5.49	-7.12 to -3.85	< 0.001	0.00%	Fixed
Pregnancy Outcomes (count)	4	196	0.17	-0.56 to 0.43	0.26	35.46%	Random

MD, mean difference; CI, confidence interval; Random, random-effect models; Fixed, fixed-effect models; NA, Not applicable.

In the POR subgroup, intraovarian PRP treatment was associated with significant improvements in AFC (MD = 0.23, 95% CI 0.16 to 0.49, P < 0.001) and AMH (MD = 0.63, 95% CI 0.45 to 0.87, P < 0.001), as well as a significant reduction in FSH levels (MD = -4.53, 95% CI -6.09 to -1.98, P < 0.001). However, no statistically significant effect on pregnancy outcomes was observed (LogRR = -0.45, 95% CI -1.97 to 0.18, P = 0.09).

In the POI/POF subgroup, PRP treatment was associated with significant improvements in AFC (MD = 1.15, 95% CI 0.77 to 2.05, P < 0.001) and AMH (MD = 0.37, 95% CI 0.22 to 0.52, P < 0.001), together with a significant decrease in FSH levels (MD = -5.49, 95% CI -7.12 to -3.85, P < 0.001). No significant difference was observed for pregnancy outcomes (LogRR = 0.17, 95% CI -0.56 to 0.43, P = 0.26).

Additional subgroup analyses based on other clinically relevant variables, including patient age, PRP preparation parameters, duration of intervention, injection dose, injection frequency, timing of intervention, and adjunctive treatments, were not feasible because the number of eligible studies and the available data within each category were insufficient for meaningful pooled analyses.

### 3.7 GRADE evaluation of evidence

**Table 4** summarizes the GRADE ratings and overall certainty of evidence, with domain-specific assessments provided in the **Supplementary Materials**. For the primary outcomes, the certainty of evidence ranged from low to moderate, largely downgraded due to imprecision, which was mainly driven by the relatively small cumulative sample size (fewer than 400 participants). For AMH, the combination of limited sample size and notable heterogeneity across studies resulted in an additional downgrade, reducing confidence in this outcome.

**Table 4:** Summary of primary outcomes with GRADE evidence assessment.

Outcomes	No. of studies	No. of patients	Mean difference [± 95% CI]	Anticipated absolute effects (95% CI)	P value	Heterogeneity assessment	Reason for downgrading
			Grade evaluation	Risk with PRP therapy		I^2^ [p value]	
AFC	5	269	MD=0.81	0.81 Higher	P<0.001	I^2^ = 41.63%	Downgraded for imprecision due to the small total sample size (<400 participants)
			95% CI [0.31 to 1.30]	(0.31 to 1.30 Higher)		[P = 0.06]	
			⊕⊕⊕⊝				
			Moderate				
AMH	4	218	SMD=0.91	0.91 Higher	P<0.001	I^2^ = 54.32%	Downgraded for imprecision due to the small total sample size (<400 participants) and high inconsistency (I² > 50%)
			95% CI [0.41 to 1.42]	(0.41 to 1.42 Higher)		[P = 0.02]	
			⊕⊕⊝⊝				
			Low				
FSH	6	363	MD=-5.72	7.03 Lower	P<0.001	I^2^ = 0.00%	Downgraded for imprecision due to the small total sample size (<400 participants)
			95% CI [-7.03 to -4.41]	(7.03 to 4.41 Lower)		[P = 0.46]	
			⊕⊕⊕⊝				
			Moderate				
E_2_	3	173	MD=26.03	26.03 Higher	P<0.001	I^2^ = 0.22%	Downgraded for imprecision due to the small total sample size (<400 participants)
			95% CI [19.53 to 32.53]	(19.53 to 32.53 Higher)		[P = 0.37]	
			⊕⊕⊕⊝				
			Moderate				

PRP, platelet-rich plasma; AMH, anti-Müllerian hormone (ng/mL); AFC, antral follicle count (count); FSH, follicle-stimulating hormone (IU/L); E2, estradiol (pg/mL). ⊕⊕⊕⊝, Moderate; ⊕⊕⊝⊝, Low.

## 4 Discussion

To our knowledge, this study is the first meta-analysis restricted to RCTs evaluating the efficacy of intraovarian PRP in women with poor ovarian reserve or ovarian insufficiency/failure. The findings suggest that PRP may improve several laboratory markers of ovarian reserve, including increased AMH, AFC, and estradiol levels, together with reduced FSH levels, indicating a potential beneficial effect on ovarian endocrine function and follicular recruitment. However, these improvements in surrogate markers did not translate into a significantly higher pregnancy rate compared with the control group. Although statistical heterogeneity was generally low for several pooled outcomes, the included studies still showed notable clinical heterogeneity in PRP preparation protocols, administration regimens, and adjunctive treatments, and most enrolled women were aged 35 years or older with relatively advanced ovarian dysfunction, which may limit the generalizability and clinical specificity of the findings. According to the GRADE assessment, the certainty of evidence ranged from low to moderate, mainly limited by small sample sizes and variability across studies. Therefore, the current evidence should be interpreted as indicating overall trends rather than providing precise guidance for individualized clinical treatment.

Although this meta-analysis demonstrated that PRP significantly improved key laboratory markers of ovarian reserve, including increases in AMH, AFC, and E2 as well as reductions in FSH, these biological gains did not translate into higher pregnancy rates. This disconnect between hormonal or follicular improvement and actual reproductive outcomes suggests that PRP may primarily influence the ovarian microenvironment or early follicular recruitment, while having a limited impact on oocyte competence, embryonic development, or implantation potential, which are the true determinants of pregnancy success (33, 34). Evidence from prior studies suggests that PRP alone is unlikely to produce meaningful clinical improvement in women with severe ovarian insufficiency, such as POI or POF (35, 36). Most patients still require additional interventions, and oocyte donation remains the most reliable option for achieving pregnancy in this population, highlighting the limited evidence supporting PRP for improving final reproductive outcomes. The interpretation of existing data is further hindered by the absence of baseline oocyte quality, embryo grading, and live birth information in many studies, making it difficult to determine whether PRP affects the key developmental steps leading to pregnancy (37, 38). Although isolated cases of menstrual recovery or pregnancy have been reported after PRP treatment in POI or DOR patients, these events may reflect individual variability rather than a consistent therapeutic effect (39). At the mechanistic level, animal studies offer some insight. Budak et al. demonstrated that intraovarian PRP injection in a rat model of ovarian injury promoted the development of preantral follicles and upregulated VEGF and IGF-1, thereby enhancing local angiogenesis and tissue repair (40). Similarly, another study reported increased cellular proliferation and tissue remodeling within the ovarian cortex after PRP administration, suggesting that PRP may modulate the early follicular microenvironment rather than reverse structural ovarian aging (41). Clinical evidence mirrors this pattern. Studies by Sadeghpour et al. and Éliás et al. report improvements in AMH, FSH, and AFC or renewed follicular activity after PRP treatment, and isolated reports describe the retrieval of euploid embryos following PRP in women with repeated aneuploid cycles (21, 42). However, these studies are small, non-randomized, and often lack appropriate controls, and improvements in clinical pregnancy or live birth rates have not been consistently observed.

PRP is a concentrated plasma product derived from autologous peripheral blood, characterized by a markedly elevated platelet content and the rapid release of numerous bioactive factors from α-granules after activation (43, 44). These include transforming growth factor-β (TGF-β), platelet-derived growth factor (PDGF), insulin-like growth factor-1/2 (IGF-1/2), epidermal growth factor (EGF), vascular endothelial growth factor (VEGF), fibroblast growth factor (FGF), and the oocyte-related growth differentiation factor-9 (GDF-9). By binding to receptors on ovarian stromal cells, theca cells, and granulosa cells, these molecules activate key intracellular pathways, including PI3K–Akt, MAPK, and Smad, thereby supporting cell proliferation, survival, differentiation, and extracellular matrix remodeling (45, 46). Angiogenic factors, such as VEGF, PDGF, and FGF, enhance local vascularization and tissue oxygenation, helping to alleviate ischemia and oxidative stress commonly associated with diminished ovarian reserve. Meanwhile, TGF-β, IGF, and various chemotactic cytokines help maintain granulosa and stromal cell function and stabilize the follicular microenvironment (47). Through this combined regulation of angiogenesis, inflammation, and cellular activity, PRP may help improve follicular recruitment and partially restore ovarian reserve–related physiological processes. In women with DOR or POI/POF, the decline in follicle number, granulosa-cell dysfunction, persistent low-grade inflammation, and immune dysregulation commonly coexist (48, 49). The anti-inflammatory components of PRP can suppress pro-inflammatory cytokines such as IL-1, IL-6, and IL-8, and modulate matrix metalloproteinase activity, thereby attenuating inflammation-related aging phenotypes and helping to restore immune homeostasis within the ovary (50). At the same time, the growth factors released from PRP improve the ovarian microenvironment and may activate residual precursor or stem-like cells, supporting early follicular recruitment and reducing follicular atresia, which is reflected clinically as an increase in AFC. As granulosa-cell function improves, AMH production rises and steroidogenesis enhances, resulting in higher E2 levels; the strengthened negative feedback on the hypothalamic–pituitary–ovarian axis subsequently lowers FSH. This produces a characteristic pattern of laboratory improvement: AMH↑, AFC↑, E2↑, FSH↓. *In vitro* studies further demonstrate that PRP promotes granulosa-cell proliferation, reduces apoptosis, improves mitochondrial activity, and dampens inflammatory signaling, supporting the concept that PRP enhances ovarian tissue repair and follicular development through multiple coordinated pathways (20, 34). Collectively, the potential mechanisms of PRP include pro-angiogenesis, immunomodulation, anti-oxidative effects, cellular activation, microenvironment optimization, and tissue regeneration, which together may account for the observed improvements in ovarian reserve–related biomarkers.

Nevertheless, improvement in these ovarian reserve–related biomarkers does not necessarily indicate a corresponding enhancement in final reproductive outcomes. Although PRP may promote angiogenesis, reduce inflammation, and improve granulosa-cell and stromal-cell function, these effects may mainly influence the ovarian microenvironment and endocrine profile rather than fully restore reproductive competence (34). Clinical pregnancy depends on multiple downstream processes, including oocyte maturity and competence, embryo developmental potential, endometrial receptivity, implantation efficiency, and the local inflammatory and endocrine milieu (51). Therefore, favorable changes in AMH, AFC, FSH, and E2 may reflect partial biological improvement without necessarily translating into meaningful gains in pregnancy outcomes (52). This distinction is clinically important because modest improvements in laboratory markers should not be interpreted as direct evidence of enhanced reproductive success. This discrepancy may be particularly relevant in women with relatively advanced ovarian dysfunction, in whom restoration of follicular recruitment or hormonal activity alone may not be sufficient to reverse impaired oocyte quality or implantation potential. Accordingly, these findings should be interpreted with particular caution in women of advanced reproductive age, especially those older than 42–43 years, because clear reproductive benefit has not yet been demonstrated in this population. At present, the available evidence is insufficient to support overly optimistic clinical expectations for PRP in this age group based solely on biomarker improvement. Future studies should further investigate whether PRP can meaningfully affect oocyte quality, embryo development, endometrial receptivity, and other mechanistic pathways linked to final reproductive outcomes.

An additional issue that should be considered when interpreting the present findings is the substantial clinical heterogeneity across the included studies. Although statistical heterogeneity was low for several pooled outcomes, important between-study differences remained in PRP preparation protocols, including platelet concentration, activator use, centrifugation procedures, injection frequency, injection volume, dosing intervals, and adjunctive interventions. These variations may have influenced treatment effects and could partly explain differences in study results. In the present review, subgroup analysis according to ovarian condition (POR vs POI/POF) was feasible and provided a partial exploration of heterogeneity. However, further subgroup analyses based on PRP protocol characteristics, such as injection dose, injection frequency, timing of intervention, and adjunctive treatments, were not possible because of the limited number of studies and insufficient reporting within each category. In addition, most included participants were women with relatively advanced ovarian dysfunction, and insufficient data were available to allow meaningful stratification according to age, disease severity, etiology, or comorbidities. As a result, the current evidence is insufficient to provide precise guidance for individualized clinical treatment or to determine whether efficacy differs meaningfully across clinically relevant subgroups. Therefore, the optimal intraovarian PRP regimen remains unclear, the generalizability of the current findings is limited, and the pooled estimates should be interpreted with caution. Moreover, from a clinical decision-making perspective, the risk–benefit balance of intraovarian PRP also remains uncertain, given the lack of clear pregnancy benefit and the limited and inconsistent reporting of safety outcomes across studies. A formal evaluation of cost-effectiveness was also not feasible, as the included studies did not provide sufficient data on treatment costs, resource utilization, or long-term clinically meaningful outcomes. Therefore, the overall clinical utility and economic value of intraovarian PRP remain to be established.

## 5 Strengths and limitations

This study has several notable strengths. First, only RCTs were included, providing a higher level of evidence than previous reviews that were largely based on observational studies. Second, the certainty of the primary outcomes was systematically evaluated using the GRADE framework, allowing a transparent and standardized interpretation of the findings. Third, the literature search was comprehensive and covered major Chinese and international databases without language restrictions, thereby reducing the risk of study omission.

Despite these strengths, several limitations should be acknowledged. First, the number of eligible trials was limited, and the overall sample size was relatively small, with most primary outcomes including fewer than 400 participants, which may have reduced the precision of the pooled estimates. Second, substantial variability existed across studies in PRP preparation and administration, including platelet concentration, activation methods, injection dose, injection frequency, treatment intervals, timing of intervention, and adjunctive treatments, which may have contributed to important clinical heterogeneity. Although subgroup analysis according to ovarian condition (POR vs POI/POF) was feasible, further stratified analyses based on PRP protocol characteristics or adjunctive interventions could not be performed because of limited study numbers and insufficient data within each category. Therefore, the optimal intraovarian PRP regimen remains uncertain. Third, some trials provided insufficient details regarding key methodological processes, such as randomization and allocation concealment, introducing potential risks of bias. Fourth, many included studies did not report comprehensive data on oocyte quality, embryonic development, live birth, or other final reproductive outcomes, limiting assessment of whether improvements in ovarian reserve markers translate into meaningful clinical benefits. In addition, outcomes related to oocyte maturity, embryo quality, implantation, endometrial receptivity, or local inflammatory status were rarely reported, limiting a more in-depth mechanistic interpretation of why improvements in ovarian reserve-related biomarkers were not accompanied by significant pregnancy benefits. Fifth, the included populations mainly consisted of women aged 35 years or older with markedly reduced ovarian reserve or ovarian insufficiency/failure, and insufficient data were available to allow meaningful stratification by age, disease severity, etiology, or comorbidities, which may limit the generalizability of the findings. Moreover, the current evidence does not allow a robust evaluation of the risk–benefit ratio of intraovarian PRP, because clinically meaningful reproductive benefits remain uncertain and safety reporting was limited across studies.

## 6 Conclusions

This meta-analysis suggests that intraovarian PRP injection may improve several laboratory indicators of ovarian reserve, including AMH, FSH, AFC, and E2, in women with poor ovarian reserve or ovarian insufficiency/failure. However, current evidence does not demonstrate a corresponding benefit in pregnancy outcomes. Given the limited number of trials, small sample sizes, and heterogeneity in PRP preparation and administration protocols, the clinical significance of these findings remains uncertain, and neither the optimal PRP regimen nor the most appropriate target population has yet been established. Larger, well-designed, and standardized RCTs are needed to clarify the efficacy, safety, subgroup-specific effects, and overall clinical value of intraovarian PRP in these populations.

## Data Availability

The original contributions presented in the study are included in the article/**Supplementary Material**. Further inquiries can be directed to the corresponding author.

## Supplementary material

Supplementary Table 1 Search strategy.
