# Supplementary material for: Efficacy of intraovarian autologous platelet-rich plasma in women with poor ovarian reserve or ovarian insufficiency: a meta-analysis of randomized controlled trials
Source: Front Endocrinol (Lausanne). 2026 May 12;17:1827041. doi: 10.3389/fendo.2026.1827041 (PMC13201205; doi:10.3389/fendo.2026.1827041)
Supplement: Supplementary Table 1 — Search strategy. [file Table1.docx]

**Search strategy**

#1 "Primary Ovarian Insufficiency" **[MeSH] OR** "Ovarian Insufficiency, Primary" **[MeSH]**

#2 "Menopause, Premature" **[MeSH] OR** "Premature Menopause" **[MeSH]**

#3 "Ovarian Failure, Premature" **[MeSH]** **OR** "Premature Ovarian Failure" **[MeSH]**

#4 "Gonadotropin-Resistant Ovary Syndrome" **[MeSH]** **OR** "Gonadotropin Resistant Ovary Syndrome" **[MeSH]**

#5 "Resistant Ovary Syndrome"

#6 "Hypergonadotropic Ovarian Failure, X-Linked" **[Title/Abstract]** **OR** "X-Linked Hypergonadotropic Ovarian Failure" **[Title/Abstract] OR** "X Linked Hypergonadotropic Ovarian Failure" **[Title/Abstract] OR** "Premature Ovarian Failure, X-Linked" **[Title/Abstract] OR** "Premature Ovarian Failure, X Linked" **[Title/Abstract]**

#7 "Fragile X-Associated Primary Ovarian Insufficiency" **[Title/Abstract] OR** "Fragile X Associated Primary Ovarian Insufficiency" **[Title/Abstract] OR** "Fragile X Premature Ovarian Failure" **[Title/Abstract]**

#8 "FMR1-Related Primary Ovarian Insufficiency" **[Title/Abstract] OR** "FMR1 Related Primary Ovarian Insufficiency" **[Title/Abstract] OR** "Primary Ovarian Insufficiency, Fragile X-Associated" **[Title/Abstract] OR** "Primary Ovarian Insufficiency, Fragile X Associated" **[Title/Abstract] OR** "Premature Ovarian Failure " **[Title/Abstract]**

#9 "poor ovarian response" **[Title/Abstract]**

#10 #1 **OR** #2 **OR** #3 **OR** #4 **OR** #5 **OR** #6 **OR** #7 **OR** #8 **OR** #9

#11 Platelet‐Rich Plasma **[MeSH]**

#12 Blood Transfusion, Autologous **[MeSH]**

#13 Thrombocyte Rich Plasma **[Title/Abstract]**

#14 plasma **[Title/Abstract]** **OR** therap* **OR** fibrin **[Title/Abstract]**

#15 PRP **[Title/Abstract]** **OR** PRF **[Title/Abstract]**

#16 platelet near **[Title/Abstract]** OR buffy layer **[Title/Abstract]**

#17 #11 **OR** #12 **OR** #13 **OR** #14 **OR** #15 **OR** #16

#18 (randomized controlled trial[pt] **OR** controlled clinical trial[pt] **OR** randomized[tiab] **OR** placebo[tiab] **OR** clinical trials as topic[mesh:noexp] **OR** randomly[tiab] **OR** trial[ti]) **NOT** (animals [mh] **NOT** (humans [mh] **AND** animals[mh]))

#19 #10 **AND** #17 **AND** #18
